# A randomized controlled trial of social cognition and interaction training for persons with first episode psychosis in Hong Kong

**DOI:** 10.3389/fpsyt.2023.1098662

**Published:** 2023-03-06

**Authors:** Panmi M. T. Lo, Simon S. Y. Lui, Colin K. M. Law, David L. Roberts, Andrew M. H. Siu

**Affiliations:** ^1^Department of Occupational Therapy, Castle Peak Hospital, Hong Kong, Hong Kong SAR, China; ^2^Department of Psychiatry, The University of Hong Kong, Hong Kong, Hong Kong SAR, China; ^3^Department of Psychiatry, University of Texas Health Science Center, San Antonio, TX, United States; ^4^Department of Health Sciences, Brunel University, London, United Kingdom

**Keywords:** social cognition, first-episode psychosis, training, rehabilitation, randomized controlled trials, follow-up study

## Abstract

Social cognitive impairment is a core limiting factor of functional recovery among persons with first episode psychosis (FEP). Social Cognition and Interaction Training (SCIT) is a group-based, manualized training with demonstrated evidence in improving social cognitive performance among people with schizophrenia. However, there are few studies on the effect of SCIT for people with FEP and for people in non-Western societies. This study evaluated the feasibility, acceptability and initial effectiveness of the locally-adapted SCIT in improving social cognitive functioning in Chinese people with FEP. The SCIT was delivered two sessions per week over a 10-weeks period, each session lasted for 60–90 min. A total of 72 subjects with FEP were recruited from an outpatient clinic and randomized to conventional rehabilitation (“Rehab”) and experimental (“SCIT and Rehab”) groups. Primary outcome measures included four social cognitive domains including emotion perception, theory-of-mind, attributional bias and jumping-to-conclusion, and secondary measures included neurocognition, social competence and quality of life. Participants were assessed at baseline, post-treatment, and 3-months post-treatment. Repeated measures ANCOVAs, with baseline scores as covariates, were used to compare the group differences in various outcomes across time. The results showed that the SCIT was well-accepted, with a satisfactory completion rate and subjective ratings of relevance in the experimental group. Moreover, treatment completers (*n* = 28) showed evidence of an advantage, over conventional group (*n* = 31), in reduced attributional bias and jumping-to-conclusions at treatment completion, lending initial support for the SCIT in Chinese people with FEP. Future research should address the limitations of this study, using more refined outcome measurements and higher treatment intensity of the SCIT.

## 1. Introduction

Successful functional recovery is an important treatment target for people with first-episode psychosis (1, 2), but remains to be challenging (3). Apart from neurocognition being an important determinant of functional recovery (4), social cognition has received increasing attention in prediction of functioning in FEP, such as work functioning (5). Social cognition is defined as a set of mental operations that underlie social interactions, including perceiving, interpreting, and generating responses to the intentions, dispositions, and behaviors of other people (6). It is a complex, multi-faceted construct encompassing several sub-domains including emotion perception, theory of mind (ToM), and attributional style/bias (7, 8). There is much evidence suggesting people with schizophrenia displayed significant impairments in emotion perception and ToM (9, 10). A subgroup with paranoid delusion demonstrated higher tendency in adopting attributional bias (11).

The close connection between poor functional outcomes, such as inability to live, work or socialize independently, and impaired social cognition in people with chronic schizophrenia (12) and FEP (5, 13) indicates that improving social cognition can potentially improve daily functioning. Moreover, people in the early-phase of psychosis were found to display less structural and functional brain changes, such as less widespread gray matter volumetric deficit, than people with chronic schizophrenia (14). This suggests that their cortical representational systems exhibit greater malleability that may optimize the treatment effect (15, 16). It is not clear if pharmacological or psychosocial interventions help to manage social cognitive problems in people with FEP (17). Considering the similarity in social cognitive impairments between people with FEP and people with established schizophrenia, it is plausible that social cognitive interventions designed for people with established schizophrenia can be applicable to people with FEP. Social Cognition and Interaction Training (SCIT) is one of the evidence-based interventions to improve social cognitive functioning (18, 19) in people with schizophrenia. SCIT is a structured and manualized group-based intervention, that addresses dysfunctional social cognitive processes, such as impaired emotion perception, theory of mind (ToM), hasty judgments, and biased social attributions (20, 21). SCIT consists of three phases that target at improving three types of social cognitive process. Phase I - emotion recognition (which addresses emotion perception dysfunction), Phase II - figuring-out situations (which addresses attributional biases and ToM dysfunction), and Phase III - integration (which involves the applications of the learned skills from Phase I and II to participants’ interpersonal problems). The efficacy of SCIT in improving social cognition and other functional recovery outcomes in people with established schizophrenia has been investigated in previous studies using non-randomized (22–24) and randomized (25) controlled trial designs.

While the evidence on the efficacy of SCIT is accumulating, there are several limitations in previous studies and the current study aims to address some of these limitations. First, this study uses a randomized controlled instead of non-randomized designs. The effects of SCIT for people with schizophrenia are promising with social cognitive gains reaching medium to large effect sizes in non-randomized studies (22, 23, 26) though these effects are weaker in randomized-design study and only small improvement in functional performance (25). There is a need to further investigate the effect of SCIT using larger sample and more rigorous design, especially for people with FEP who are believed to have greater brain plasticity to benefit from treatment. To-date, only one previous study (27) has investigated the effect of SCIT on FEP. This study did not have any control group, and only compared pre- and post-intervention outcomes. The preliminary results suggested that people with FEP improved in emotion perception and social/occupational functioning after receiving SCIT (27). Second, most previous studies of SCIT are conducted in U.S., the effect of SCIT for patients of non-Western culture is not well understood (28–30). In the two studies conducted for Chinese samples with schizophrenia, the researchers made minimal adaptation to SCIT to examine potential cultural differences (29, 30). There is initial evidence suggesting cultural differences in social cognitive processing in healthy people. For instance, the Chinese and the Western populations differ in social cognition, such as self-relevance processing (31) and perspective taking mechanism (32–34). Individual’s vocabulary knowledge may also impact their performance in ToM tasks (35, 36). Taken together, it is necessary to have a culturally-adapted SCIT (37) to investigate its effects on social cognition in the Chinese setting. Lastly, previous studies usually use social cognition and functional measures as outcome measures. However, people with schizophrenia and FEP both demonstrated impaired performance in a range of neurocognitive tasks (38, 39). Considering the medium-range correlation between neurocognition and social cognition (13, 40, 41), it is possible that social cognitive training may remediate neurocognition (42, 43) on top of social cognition. In this study, we would include measures of neurocognition as a secondary outcome measure.

This study aimed to examine the feasibility, acceptability and initial effectiveness of a culturally-adapted SCIT version on social cognition and neurocognition in people with FEP in a non-Western context, using the robust method of randomized-controlled trial. Following Horan & Green (18)‘s recommendation, we administered SCIT as an additional intervention to the conventional rehabilitation programs. We hypothesized that the SCIT group (which received both SCIT and conventional rehabilitation programs) would show a higher level of improvement over time in social cognition, neurocognition and functioning, when compared with the control group (which received conventional rehabilitation program). The primary outcome of this study was social cognition, and the secondary outcomes were neurocognition, social competence and quality of life.

## 2. Method

### 2.1. Participants

Participants were outpatients recruited from an early psychosis intervention clinic in Hong Kong. The inclusion criteria were: (1) ICD-10 diagnoses of schizophrenia, schizoaffective disorder or unspecified psychosis, (2) first-episode psychosis with a duration of illness no longer than 2 years, (3) aged 18 to 45 years old, and (4) able to understand spoken and written Chinese sufficiently to follow testing procedures and participate in SCIT. The exclusion criteria were: (1) history of relapse of psychosis, (2) intellectual disability, (3) history of traumatic brain injury or neurological disorder, (4) history of alcohol or substance abuse in the past 6 months, and (5) history of drug-induced psychosis. To further minimize the possible confounding effects of medication, outpatients who had a planned change of medication in the coming 3 months at time of recruitment were excluded from this study. Moreover, we also excluded those outpatients who received high dose benzhexol (i.e., 12 mg/days or above). The inclusion and exclusion criteria were confirmed by retrieving information from hospital medical records. The clinical diagnosis was ascertained by qualified psychiatrists, supplemented by review of medical records.

Previous studies of SCIT for people with schizophrenia reported effect sizes ranging from 0.29 to 0.50 across different types of social cognition (22, 23). Based on the assumptions of α = 0.05, an estimated effect size of 0.40, and a 10% attrition rate (23, 25), we estimated 32 participants in each group were needed to achieve power of 0.80.

### 2.2. Procedures

Approval from Research Ethics Committee of the Hospital Authority was obtained. All potential participants were approached by clinic staff. Those who agreed to join the study and who matched the inclusion and exclusion criteria were recruited and randomized into either the control or the experimental group using simple randomization, flipping a coin method. Written informed consent was obtained from all participants before the start of data collection. At baseline, participants’ clinical symptoms and IQ were assessed by psychiatrists and a trained research assistant, respectively. Occupational therapists, blinded to the group assignment, administered the outcome measures on social cognition, neurocognition and social competence at pre-treatment, post-treatment and 3-month post-treatment.

### 2.3. Treatment conditions

The control group received the conventional rehabilitation (Rehab) program in the participating clinic. The conventional programs covered elements like: (1) vocational/study goal setting, (2) career/study choice exploration, (3) various life skills enhancement training such as stress management, (4) work-related social skills training, to (5) job acquisition skills training. The conventional rehabilitation programs were delivered by qualified occupational therapists, guided by practice manuals and service model of the Hong Kong Hospital Authority. These programs were selected and implemented to participants based on individuals’ needs as part of standard service of the participating clinic. These programs ran 1 to 2 sessions per week on average within the 10-week review period. On the other hand, the intervention group received both SCIT and the conventional rehabilitation programs (i.e., SCIT + Rehab). Both groups received other routine interventions offered in the early psychosis clinic including pharmacological intervention and case management.

In this study, the SCIT-Hong Kong version was translated and modified from the original English version. The structure and session flow of this version was the same as the original version, with modifications on the social stimuli used in the training exercises. All the training photos and videos were produced with Chinese people as actors to ensure cultural adaptation (44). Therefore, the social stimuli fitted well to the local contexts (e.g., replacing “hamburgers” with Asian Food, replacing “mailroom of an office” with “storeroom,” and incorporating a wider range of interpersonal scenarios at the workplace such as misunderstandings with colleagues or guessing intentions of a work supervisor). Terminologies used in SCIT such as “jumping to conclusion,” “ambiguous social situations” were carefully translated into the Chinese language. The original version of the SCIT required subjects to be highly interactive and actively involved in group discussion. Considering the learning culture among Chinese, our version of the SCIT used PowerPoint and participants’ manuals to guide and facilitate discussion. In this study, the SCIT comprised 19 sessions, delivered in 10 weeks (two sessions per week). Each group consisted of four to eight participants, led by two experienced clinicians. Each session lasted for 60–90 min. Like the practice of other routine programs, participants were reached by phone to remind attendance on the day before each session. The clinicians (PL and another clinician) had Master degree and more than 5 years of experience in group-based training. PL also received intensive training by developer of SCIT (DLR).

### 2.4. Instruments

#### 2.4.1. Clinical profile

Participants’ psychiatric symptoms and estimated intelligence were measured. Qualified psychiatrists administered the Positive and Negative Syndrome Scale [PANSS; (45)], the Montgomery-Asberg Depression Rating Scale [MADRS; (46)] and the Social and Occupational Functioning Assessment Scale [SOFAS; (47)] through a structured interview. The Chinese version of the arithmetic, similarities and digit span subtests of the Wechsler Adult Intelligence Scale-Revised [WAIS; (48)] was administered by trained research assistants to estimate participants’ IQ, as IQ is a confounding variable of cognitive function measures.

#### 2.4.2. Feasibility and acceptability of the Chinese version of the SCIT

The feasibility of SCIT was explored based on the persistence rate at treatment end and attendance rate of experimental group. The acceptability was evaluated using questionnaire to gather participants’ feedback on the SCIT. Immediately after completion of SCIT, participants were invited to complete a feedback questionnaire in which they rated five aspects of SCIT, including the perceived usefulness of the training in understanding emotions/thoughts of other people or in getting along with others; the usefulness of the participant workbook in facilitating the learning of content; and the practicability of the training. The sixth question asked participants to rate the overall satisfaction level. Each question was answered using a 5-point Likert scale ranging from 1 (totally disagree) to 5 (totally agree). The questionnaire was given to participants in the last session of SCIT by a therapy assistant, without the presence of the clinicians who provided the SCIT intervention.

#### 2.4.3. Social cognitive measures

Emotion perception was assessed with the Chinese Facial Emotion Identification Test (C-FEIT) (49). The C-FEIT requires participants to perceive emotions from 21 different photos, depicting happy, sad, disgusted, angry, fearful, surprise and neutral emotion. The C-FEIT score could range from 0 to 21, with higher score indicating better facial emotion identification ability. The mean C-FEIT score of schizophrenia sample was 13.63 in a validation study in a Chinese setting (49). In this study, C-FEIT demonstrated satisfactory test–retest reliability (ICC = 0.85), internal consistency (Cronbach’s α = 0.78) and low to medium correlations with neurocognitive measures (r ranges from 0.29 to 0.45) (49). The Chinese Social Cognitive Screening Questionnaire (C-SCSQ), validated from original English version SCSQ (50) was used to assess participants’ ToM ability (range 0–10, with higher score indicating better performance, mean score of schizophrenia sample = 6.32), attributional bias (range 0–5, with lower score indicating less hostile attributional bias, mean score of schizophrenia sample = 2.93) and jumping to conclusion bias (range 0–4, with lower score indicating less JTC bias, mean score of schizophrenia sample = 1.68) (49). The C-SCSQ requires participants to infer intentions of the characters described in 10 vignettes of different social situations. The C-SCSQ has been validated in the Chinese setting and subscales of C-SCSQ were found to have satisfactory test–retest reliability (ICC ranges from 0.76 to 0.85), known-group validity (d ranges from 1.26 to 3.27) and low to medium correlations with neurocognitive measures (r ranges from 0.25 to 0.34) (49). On top of these, the psychometric properties of SCSQ have also been tested and supported in another culture with Japanese sample (51). In this study, the ToM and PAS subscales of SCSQ showed significant low to medium correlations with common social cognitive measures, the Hinting Task (52) (r = 0.52) and AIHQ (11) (r ranges from 0.34 to 0.47) respectively, supporting its criterion-related validity. SCSQ total score highly discriminated patients and healthy controls, supporting its discriminate validity (51).

#### 2.4.4. Neurocognitive measures

The MATRICS Consensus Cognitive Battery (MCCB) (53) was used to assess participants’ cognitive performance, including speed of processing, attention/vigilance, working memory, verbal learning, visual learning and reasoning and problem solving. The MCCB is a validated and commonly used neurocognitive assessment battery for people with schizophrenia and has been found to have acceptable to good test–retest reliability (ICC = 0.68 to 0.85), small practice effect with no noticeable ceiling effect and low to medium correlations with functional outcomes (53).

#### 2.4.5. Social competence and quality of life

Social competence and quality of life are regarded as secondary outcomes measures in this study, as SCIT could have an indirect or longer-term effects on these variables (54). The Personal-Social Development Self-efficacy Inventory (PSDSEI; (55)) is a self-rated instrument that assesses subjective competence in handling interpersonal social situations among adolescents. Participants completed the subscales on understanding others, cooperation, thinking and expression skills, and management of stress and emotion to measure their change in social competence after treatment. The short version of the World Health Organization Quality of Life instrument (WHOQOL) (56) was used to assess participants’ quality of life and general wellbeing.

### 2.5. Statistical analysis

Demographics, baseline clinical characteristics, IQ and cognitive functions of the two groups were compared using chi-square, and independent t-test or Mann–Whitney U test. Repeated measures analysis of variance (ANOVAs) were used to examine both the interaction effects and within group differences, at the pre-treatment and post-treatment time-point, as well as after 3-month follow-up, in terms of the primary and secondary outcomes. To account for potentially confounding effect of baseline values of the outcome in RCT (57, 58), ANCOVAs were conducted using baseline scores as covariates. Corrections for multiple comparisons were also performed. ANOVA and ANCOVAs were conducted firstly for all participants who completed assessments at post-treatment and at follow-up (i.e., modified intention-to-treat (ITT) analysis) and were repeated for those participants who were “treatment completers” (i.e., completer analysis). “Treatment completers” was defined as those who had at least 50% attendance in each of the three phases of SCIT (25).

## 3. Results

### 3.1. Sociodemographic and clinical profiles of participants

A total of 72 participants met the selection criteria and were randomized into either SCIT+Rehab group (*n* = 39) or Rehab group (*n* = 33). The mean age of the participants was 25.2 (SD = 6.3) years old and the mean age at onset of psychosis was 24.4 (SD = 7.4) years old. The mean PANSS scores were low across the three scales, suggesting that the participants had few psychiatric symptoms. The mean and mode of MADRS were 2.9 and 2.0 respectively, which indicate that depressive symptoms are uncommon among participants (59). The participants had a mean score of 70.2 (SD = 12.5) on the SOFAS, indicating good functioning in the community. All participants were prescribed antipsychotic medication at the time of recruitment. The groups were receiving comparable doses of antipsychotic medications at baseline. Only a few participants in both groups were receiving low dose benzhexol (*n* = 5 in SCIT+Rehab and *n* = 8 in Rehab).

**Figure 1:**
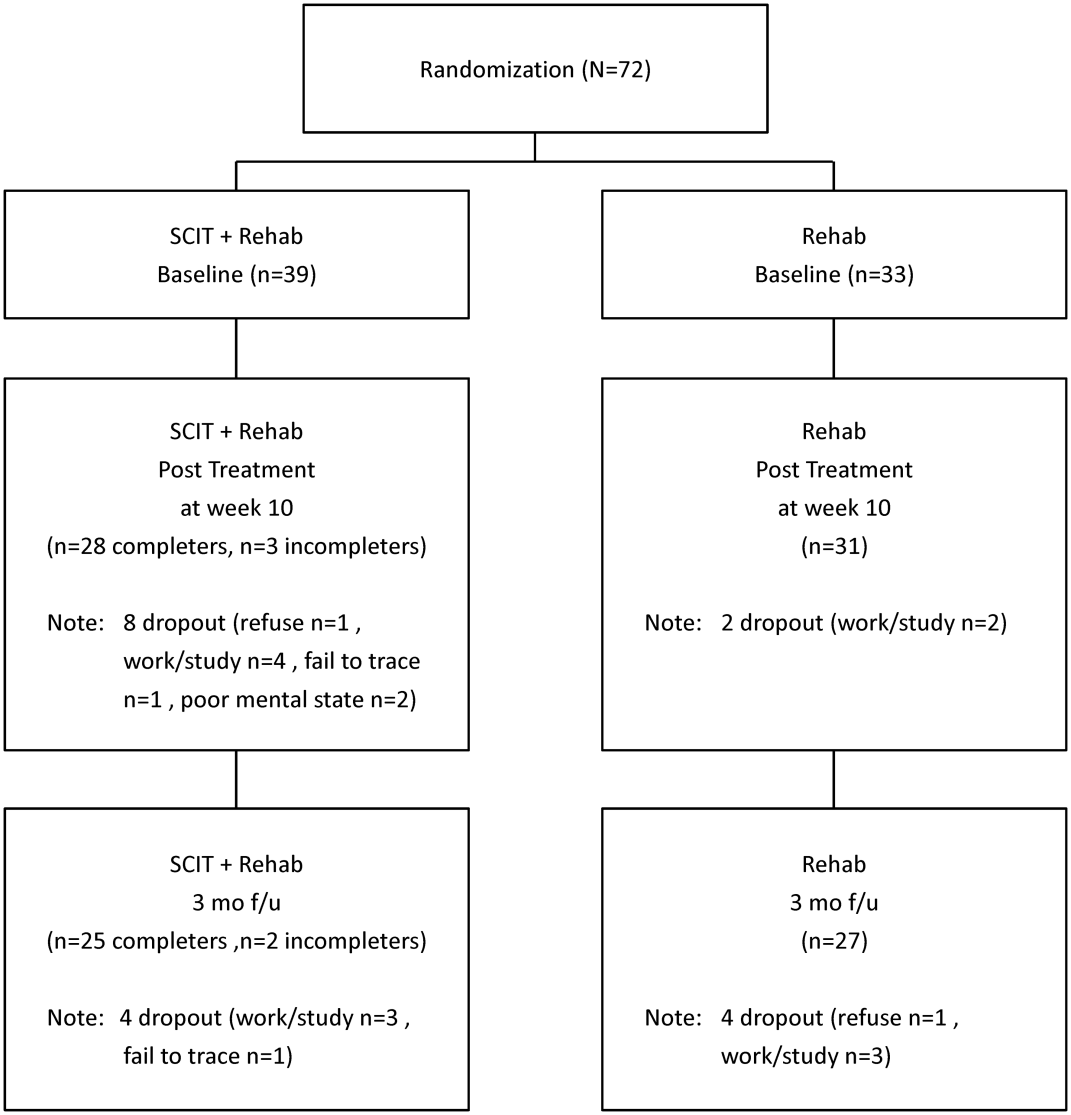
Consort diagram.

Among the 39 participants randomized to SCIT+Rehab, 31 completed post-treatment assessment (28 were “treatment completers” and 3 were “non-completers”) and 27 completed follow-up assessment (25 were “treatment completers” and 2 were “non-completers”). Among the 33 participants randomized to Rehab, 31 completed post-treatment assessment and 27 completed follow-up assessment (Figure 1). The SCIT+Rehab group and the Rehab group did not differ significantly in any demographic and clinical variables as well as outcome variables at baseline (Table 1). There were no significant differences in attrition rates between the two groups at post treatment (χ^2^ = 3.12, *p* = 0.10) and at follow up (χ^2^ = 1.5, *p* = 0.28).

**Table 1:** Demographic and clinical characteristics at T1 (*N* = 52).

Characteristics	SCIT + Rehab (*n* = 25)	Rehab (*n* = 27)	*p*
Age, mean (SD)	25.1 (5.2)	26.4 (7.5)	0.720^a^
Education in years, mean (SD)			
Time since psychosis onset, mean (SD) in mths	19.9 (22.9)	20.7 (23.3)	0.905
Male sex	*n* = 12, 48.0%	*n* = 11, 40.7%	0.780^b^
IQ, mean (SD)	97.4 (19.1)	93.4 (21.7)	0.503
Primary diagnosis			
Schizophrenia	*n* = 22, 88.0%	*n* = 23, 85.2%	0.542^a^
Unspecified psychosis	*n* = 2, 8.0%	*n* = 3, 11.1%	
Acute and transient psychosis	*n* = 1, 4.0%	*n* = 0, 0.0%	
Schizoaffective disorder	*n* = 0, 0.0%	*n* = 1, 3.7%	
Secondary diagnosis			
Depression	*n* = 4, 16.0%	*n* = 4, 14.8%	0.368^a^
Anxiety	*n* = 1, 4.0%	*n* = 0, 0.0%	
Suspected pervasive developmental disorder	*n* = 0, 0.0%	*n* = 1, 3.7%	
PANSS_P	9.4 (3.9)	8.1 (2.5)	0.156^a^
PANSS_N	12.1(6.2)	9.4 (3.4)	0.082^a^
PANSS_G	21.5 (5.7)	19.6 (4.3)	0.174
MADRS	3.8 (7.8)	2.6 (3.7)	0.473
SOFAS	68.4 (11.8)	71.0 (12.9)	0.454
Chlorpromazine equivalents	448.7 (256.0)	439.7 (279.6)	0.908
Taking Artane	*n* = 5, 20.0%	*n* = 8, 29.6%	
Artane dosage, M (SD) in daily mg	5.6 (3.8)	4.8 (2.4)	0.628

a Mann–Whitney U.

b Pearson chi-square OR Fisher’s Exact Test, only the data from participants who were treatment completers (SCIT + Rehab group) and who completed assessment (Rehab group) at follow-up is included.

### 3.2. Feasibility and acceptability of SCIT

For feasibility issues, we estimated the persistence rate at completion of SCIT (i.e., T2) and the average attendance rate. Among the 39 participants randomized to SCIT+Rehab, 8 dropped out of the study due to various reasons, like work/study (*n* = 4), poor mental state (*n* = 2), refuse to continue (*n* = 1), unknown reason (*n* = 1) (Figure 1). The persistence rate at treatment completion was 79.5% (31/39). Over 70 % (71.8%) of participants were “treatment completers.” The average attendance rate among treatment completers was 75% which was similar to the attendance rate of 69% reported in a pilot study of the SCIT for people with early psychosis (27).

The acceptability of the SCIT was explored using a satisfaction survey. The results showed that participants’ responses were positive (Table 2). Participants agreed that SCIT was practical (M = 4.13, SD = 0.57) and were highly satisfied with the training, with a mean of 4.23 SD = 0.68 on a 5-point scale. Among the three aspects on usefulness of SCIT, participants found SCIT to be most helpful in improving their understanding about the thoughts of other people (M = 4.13, SD = 0.51), followed by getting along with people (M = 4.10, SD = 0.76) and in improving their understanding of emotions of other people (M = 3.97, SD = 0.67). Most (93.3%) participants agreed or totally agreed that SCIT had helped them in understanding thoughts of other people.

**Table 2:** Feedback on SCIT from participants.

Item	Responses						
	“1” Totally disagree	“2” Disagree	“3” Neutral	“4” Agree	“5” Totally agree	M	S.D.
	% of respondents						
1. To what extent do you agree the content was practical?	0	0	10.0	66.7	23.3	4.13	0.57
2. To what extent do you agree the training has helped you in understanding emotions of other people?	0	0	23.3	56.7	20.0	3.97	0.67
3. To what extent do you agree the training has helped you in understanding thoughts of other people in different social situations?	0	0	6.7	73.3	20.0	4.13	0.51
4. To what extent, do you agree the training has helped you in getting along with people?	0	0	23.3	43.3	33.3	4.10	0.76
5. To what extent, do you agree the participant booklet has helped you in learning the course content?	0	0	13.3	70.0	16.7	4.03	0.56
6. On the whole, are you satisfied with the training?	0	0	13.3	50.0	36.7	4.23	0.68

### 3.3. Social cognition outcomes

All Group × Time interaction effects at post treatment and 3-month follow-up failed to reach statistical significance using modified ITT analysis. From results of completer analysis using ANCOVAs, after controlling for baseline scores, the Group × Time (2 × 2) interaction effect at the post treatment were statistically significant for hostile attributional bias (*F* = 4.84, *p* = 0.03) and jumping to conclusion tendency (*F* = 5.08, *p* = 0.03). At 3-month follow up, there was significant Group × Time (2 × 3) interaction effect in hostile attributional bias only, however the effect did not maintain after controlling for baseline score. For within-group differences, only the treatment completers in SCIT + Rehab group had a significantly lower score in attributional bias at post-treatment compared with baseline though the effect size was small (η_p_^2^ = 0.16, *p* = 0.03), whereas the Rehab group displayed a trend of worsening attributional bias, contributing to the interaction effect. Similar trends of reducing jumping to conclusion among treatment completers and increasing jumping to conclusion in comparison group were observed. There were no significant interaction effects or within-group differences in emotion perception and theory-of-mind measures at both post treatment and 3-month follow-up. Participants’ social cognitive functioning (primary outcome) at baseline, post-treatment and 3-month follow-up are summarized in Tables 3 and 4 respectively.

**Table 3:** Comparison of social cognitive and neurocognitive measures between SCIT + Rehab completers and Rehab group at T1 and T2.

Measures	SCIT + Rehab (*N* = 28)		Rehab (*N* = 31)		Group × Time interaction (2 × 2 mixed ANOVA)		
	M (S.D.)	Within-group contrast	M (S.D.)	Within-group contrast	F	*p*	Partial eta squared
Facial Emotion Identification Test (FEIT)							
Baseline	15.57 (3.4)	NS	15.29 (2.9)	NS	0.21	0.65	0.00
Post-treatment	16.04 (2.8)		15.26 (3.3)		(0.78)	(0.38)	(0.01)
Social Cognition and Screening Questionnaire (SCSQ)							
Theory of Mind (ToM)							
Baseline	6.75 (1.3)	NS	6.77 (1.6)	NS	0.49	0.49	0.01
Post-treatment	6.46 (1.5)		6.81 (1.2)		(1.00)	(0.32)	(0.02)
Attributional Style							
Baseline	1.39 (1.07)	ES = 0.16*	1.13 (1.02)	NS	5.87	0.02*	0.09
Post-treatment	1.02 (0.82)		1.34 (1.17)		(4.84)	(0.03*)	(0.08)
Jump-to-Conclusion							
Baseline	2.69 (0.61)	NS	2.63 (0.87)	NS	3.28	0.08^+^	0.05
Post-treatment	2.54 (0.40)		2.82 (0.68)		(5.08)	(0.03*)	(0.08)
MCCB							
Speed of Processing							
Baseline	36.87 (11.7)	NS	36.26 (12.9)	NS	0.54	0.47	0.01
Post-treatment	38.61 (11.4)		39.26 (11.8)		(0.50)	(0.48)	(0.01)
Attention/Vigilance							
Baseline	44.21 (10.9)	NS	40.94(12.8)	NS	0.25	0.62	0.00
Post-treatment	43.54 (12.1)		41.29 (13.2)		(0.07)	(0.80)	(0.00)
Working memory							
Baseline	43.61 (11.7)	ES = 0.11 ^+^	46.71. (10.0)	NS	2.24	0.14	0.04
Post-treatment	46.71 (11.6)		45.48 (12.6)		(1.30)	(0.26)	(0.02)
Verbal Learning							
Baseline	41.43 (7.8)	NS	40.48 (9.9)	ES = 0.12^+^	0.31	1.06	0.02
Post-treatment	43.64 (8.5)		46.19 (14.2)		(0.89)	(0.35)	(0.02)
Reasoning & Problem Solving							
Baseline	40.57 (13.0)	NS	37.90 (12.1)	ES = 0.11^+^	0.02	0.96	0.00
Post-treatment	43.89 (12.1)		41.35 (10.2)		(0.22)	(0.64)	(0.00)
Visual Learning							
Baseline	42.82 (13.6)	NS	41.94 (10.4)	ES = 0.11^+^	0.02	0.90	0.00
Post-treatment	45.71 (11.3)		44.55 (10.8)		(0.09)	(0.77)	(0.00)

ES = effect size. NS = not significant. Baseline score added as covariate shown in blanket.

* *p* < 0.05;

+ *p* < 0.1.

**Table 4:** Comparison of social cognitive and neurocognitive measures between SCIT + Rehab completers and Rehab group at T1, T2 and T3.

Measures	SCIT + Rehab (*N* = 25)		Rehab (*N* = 27)		Group × Time interaction (2 × 3 mixed ANOVA)		
	M (S.D.)	Within-group contrast	M (S.D.)	Within-group contrast	F	*p*	Partial eta squared
Facial Emotion Identification Test (FEIT)							
Baseline	15.48 (3.6)	NS	15.15 (2.8)	NS	0.14	0.84	0.00
Post-treatment	15.96 (2.8)		15.15 (3.6)		(0.36)	(0.70)	(0.01)
Follow-up	15.64 (3.3)		15.15 (3.8)				
Social Cognition and Screening Questionnaire (SCSQ)							
Theory of Mind (ToM)							
Baseline	6.64 (1.3)	NS	6.67 (1.7)	NS	0.40	0.67	0.01
Post-treatment	6.44 (1.5)		6.81 (1.1)		(0.60)	(0.55)	(0.01)
Follow-up	6.64 (1.5)		7.04 (1.7)				
Attributional Style							
Baseline	1.48 (1.0)	ES = 0.15*	1.14 (1.1)	NS	3.37	0.04*	0.06
Post-treatment	1.02 (0.8)	T1 > T2*	1.41 (1.2)		(2.35)	(0.10)	(0.05)
Follow-up	1.12 (1.2)		1.37 (1.3)				
Jump-to-Conclusion							
Baseline	2.68 (0.6)	NS	2.60 (0.9)	NS	2.14	0.12	0.04
Post-treatment	2.55 (0.4)		2.86 (0.7)		(2.33)	(0.10)	(0.05)
Follow-up	2.49 (0.6)		2.77 (1.2)				
MCCB							
Speed of Processing							
Baseline	36.08 (12.0)	ES = 0.16	36.22 (13.8)	ES = 0.19	0.23	0.79	0.01
Post-treatment	37.96 (11.6)	T1 < T3*	39.22 (12.5)	T1 < T3*	(0.26)	(0.77)	(0.01)
Follow-up	40.00 (11.3)		41.33 (13.2)				
Attention/Vigilance							
Baseline	43.32 (11.1)	NS	42.15 (12.2)	NS	0.07	0.93	0.00
Post-treatment	43.60 (12.5)		42.89 (12.9)		(0.03)	(0.97)	(0.00)
Follow-up	44.96 (9.3)		44.63 (13.5)				
Working memory							
Baseline	42.44 (11.7)	NS	45.81 (9.3)	NS	1.11	0.33	0.02
Post-treatment	45.96 (11.9)		44.89 (13.1)		(0.61)	(0.55)	(0.01)
Follow-up	44.84 (12.7)		45.70 (13.6)				
Verbal Learning							
Baseline	41.52 (8.1)	ES = 0.12	40.78 (10.5)	ES = 0.09	0.52	0.60	0.01
Post-treatment	42.48 (8.1)	T1 < T3^+^	45.15 (14.0)	T1 < T3^+^	(0.44)	(0.65)	(0.01)
Follow-up	45.84 (10.8)		46.78 (13.2)				
Reasoning & Problem Solving							
Baseline	40.00 (13.5)	ES = 0.18	37.89 (12.4)	ES = 0.14	0.28	0.76	0.01
Post-treatment	43.88 (12.6)	T1 < T3*	41.00 (10.6)	T1 < T3*	(0.80)	(0.45)	(0.02)
Follow-up	47.44 (12.8)		43.19 (10.2)				
Visual Learning							
Baseline	42.16 (14.0)	NS	41.74 (11.0)	ES = 0.11	0.10	0.89	0.00
Post-treatment	44.92 (11.7)		45.03 (11.4)	T1 < T2^+^	(0.11)	(0.88)	(0.00)
Follow-up	46.76 (13.8)		45.70 (12.7)				

ES = effect size. NS = not significant. Baseline score added as covariate shown in blanket.

* *p* < 0.05;

+ *p* < 0.1.

### 3.4. Neurocognitive outcomes

All Group x Time interaction effects at post treatment and 3-month follow-up failed to reach statistical significance in both modified ITT and completers analyses. There were, however, significant main effect on several neurocognitive domains at follow-ups in both SCIT + Rehab and Rehab group with statistically significant improvements in speed of processing (modified ITT analysis: SCIT + Rehab: *p* = 0.02, Rehab: *p* < 0.01; completer analysis: SCIT + Rehab: *p* = 0.02; Rehab: *p* < 0.01), reasoning and problem solving (modified ITT analysis: SCIT + Rehab: *p* < 0.01; Rehab: *p* < 0.01; completer analysis: SCIT + Rehab: *p* = 0.02, Rehab: *p* = 0.02) domains across both groups, as well as trend-level improvement in visual learning (modified ITT analysis: SCIT + Rehab: *p* < 0.01; Rehab: *p* = 0.06; completer analysis: SCIT + Rehab: *p* = 0.07, Rehab: *p* = 0.09) in both groups. Participants’ neurocognitive functioning at baseline, post-treatment and 3-month follow-up are summarized in Tables 3 and 4 respectively.

### 3.5. Social competence and quality of life

The Group x Time interaction effect across all social competence subscales and WHOQOL at both post-treatment and 3-month follow up all failed to reach statistical significance using both modified ITT and completers analyses. There was only one significant within-group difference across variables. The SCIT + Rehab group had higher score in one of the social competence subscales (“understanding others”) at follow-up compared with baseline (modified ITT analysis: η_p_^2^ = 0.15, *p* = 0.04; completer analysis: η_p_^2^ = 0.14, *p* = 0.03).

### 3.6. *Post-hoc* covariate analyses

As there were no significant group differences in the baseline demographic and clinical symptoms, these variables were not included as covariates in the analysis. To examine possible dose–response effects in SCIT treatment group, the attendance rate was included as a covariate in ANCOVA analyses of within-group differences in social cognitive performance. We found significant Time × Attendance effects on attributional bias at post treatment (*F* = 10.1, *p* < 0.01) and at three-month follow-up (*F* = 6.25, *p* < 0.01).

## 4. Discussion

This study evaluated the feasibility, acceptability and the initial effectiveness of the Chinese version of the SCIT in enhancing social cognitive performance among a group of relatively high-functioning first-episode psychosis outpatients in Hong Kong. To our knowledge, this study is the first randomized clinical trial of SCIT for patients with first-episode psychosis. After cultural adaptations on the training content, the SCIT is suitable and is well-tolerated in first-episode psychosis outpatients in Hong Kong. Participants rated the training as highly relevant in enhancing their social understandings and functioning. Despite participants’ positive accounts, our findings did not show social cognitive gains in most of the social cognitive domains using social cognitive scales, except that SCIT completers showed an advantage over the comparison group in reducing hostile attributional bias and jumping to conclusion tendency after treatment. This effect did not persist at follow-up. In short, our findings support the feasibility and acceptability of the SCIT among FEP in the Chinese setting and suggest that attributional bias may reduce after SCIT.

The persistence rate and attendance rate of the participants support the feasibility of SCIT for individuals with FEP in Hong Kong. The participants gave high ratings on usefulness of SCIT in particular in enhancing their understandings towards other people’s thoughts and in getting along with other people. These support that SCIT is well-accepted and is valued in our FEP sample. Despite good treatment compliance and perceived usefulness among our participants, minimal effect of SCIT on social cognitive improvements could be detected using social cognitive scales. Among the studied social cognitive domains, the lack of effect of SCIT on enhancing emotion perception is unexpected, and this result is contrary to previous evidence showing substantial improvements in emotion perception in patients with established schizophrenia after social cognitive intervention (19). One possible explanation may be related to the relatively preserved emotion perception ability among our high-functioning early schizophrenia sample. The distributions of our sample’s FEIT performance is highly left-skewed, with above-half of our sample (54%) performing within the normative range at baseline (49). Thus, the limited impact of SCIT on emotion perception performance may be attributable to ceiling effect in the baseline of FEIT. Notably, some research suggests that deficits in emotion perception among FEP or early psychosis may be less consistent than that seen in chronic schizophrenia (60), despite the support from prior studies suggesting consistent emotion perception deficit in FEP or early psychosis. In one review, longer duration of illness was a significant moderator of treatment effect on emotion perception after social cognitive intervention (61). It would be interesting for future studies to examine if duration of illness, degree of impairments or other clinical characteristics would moderate the treatment effect considering the inadequate and inconsistent findings reported in existing studies (25, 61, 62). Another possible reason for the small effect sizes across social cognitive domains may be related to our outcome measurements. The FEIT and the SCSQ used in our study were validated locally with patients with schizophrenia of around middle-aged (49). However, the suitability of these measures for use in clinical trials among FEP has not been thoroughly examined. One recent study concluded that only one social cognitive measure, the Hinting Task, was appropriate for use in people with early psychosis (63) while several measures were recommended for middle-aged persons with schizophrenia in the SCOPE study (44). The Hinting Task bears its own limitation, including poor test–retest reliability in an early psychosis sample (63) and high chances of having ceiling effects in community samples (25, 64). There is a need to develop more refined outcome measurements for the SCIT, such as self−/informant-report or ecological social cognition measures (65, 66) to capture social cognitive processes which are typically unfold in daily life (67, 68) that cannot be reflected in traditional scales.

This study observed a small effect of reduction in attributional bias and jumping to conclusion tendency among SCIT completers, as compared with those receiving conventional rehabilitation only. Therefore, the findings lend initial support that attributional bias among people with FEP may be amendable through the SCIT. Attributional bias describes how individuals make sense of the causes of positive and negative social events encountered in daily life. Attributional bias, together with the tendency of jumping to conclusion, may result in perceiving and concluding more hostile intention from other people in negative social events (69), adversely affecting social behaviors (70). Attributional bias may be a particularly important treatment target among FEP because of its links with development of paranoia (71), recurrent relapses, and difficulties in occupational functioning (5). Our sample attained a lower mean score in attributional bias than that of the schizophrenia samples with longer duration of illness in previous studies using the SCSQ measure (49, 51). This may suggest a worsening trend of bias with increasing duration of illness and supports the value of identifying effective interventions for reducing attributional bias among FEP in their early phase of illness. Furthermore, jumping to conclusion in people with FEP is associated with more implausible delusional subtypes (72) and may predict a worse prognosis (73). Most previous intervention studies in established (chronic) schizophrenia samples failed to find an effect in attributional bias (61, 74), except a trend level of improvement in one study (25); or did not evaluate the effect on jumping to conclusion. It is encouraging to find that SCIT could reduce attributional bias and jumping to conclusion tendency, even though the change is not sustained at follow-up in our study. In view of our findings, future research should consider longer training sessions, because a significant dose–response effect on attributional bias has been found in previous (25) and our studies. Booster sessions in social cognitive intervention may also be needed (18).

The study did not find any interaction effects in all secondary measures. There are limited compelling evidence of social cognition treatment on secondary outcomes observed in previous well-designed studies among schizophrenia (25, 75) despite the extensive support on associations between social cognition and functional measures (76). This urges for more work in identifying effective treatment contents or protocol to bring impact on both social cognitive tasks and secondary outcomes. One potential reason for the lack of interaction effect in our study may be related to the nature of comparison group which received intensive rehabilitation like work or social skills training on a regular basis. This could greatly reduce the effect sizes in secondary outcomes. However, we found a small within-group effect that SCIT completers rated themselves as having higher competence in understanding others in social situations after treatment. This is consistent with their high level agreement on usefulness of SCIT in understanding about the thoughts of other people in different social situations, suggesting that the SCIT could bring impact to participants’ self-perceived social competence.

Another observation is that the total sample had significant improvements in some neurocognitive domains, but not any of the social cognitive domains, at follow-up. This is consistent with previous findings on selective improvements in some cognitive domains over time, with or without treatment, among individuals with FEP (77, 78), which may suggest different pathophysiological mechanism underlying deficits in different mental functions (79). On the other hand, the results of the few studies on social cognitive performance were mixed (60). The stability of social cognitive performance across different phases of schizophrenia remains controversial. Future studies can use a follow-up design to address this issue.

Our study has several limitations. First, we did not employ intention-to-treat (ITT) analysis. Although we found no significant differences in most of the baseline clinical, demographic or outcome variables between participants retained for analysis and those who dropped out, we agree ITT is the best approach to give an unbiased estimate of treatment effect. However, ITT is not possible in our study as we could not make contact with participants who dropped out or did not attend the re-assessments at the post-intervention and follow-up. Second, the participants were not blinded to treatment allocation which may induce bias, though the bias on the primary social cognitive measures should be minimal as the measures are objective performance-based measures. Third, we used the same social cognitive measures at baseline, post-treatment and follow-up that may result in potential practice effect. Although we did account for this by controlling participants’ baseline performances and by examining the Group x Time interaction effect as our main analysis, we suggest future clinical trials can develop and use alternate forms for social cognitive measures.

To conclude, our study supports the feasibility and acceptability of the Chinese version of SCIT for use in patients with FEP in Hong Kong. Attributional bias may be amendable through the SCIT. Further research should replicate the current research design, use a larger sample, more refined outcome measures of social cognition and function. Longitudinal design using a longer follow-up period, and intention-to-treat (ITT) analysis will strengthen the study.

## Data availability statement

The raw data supporting the conclusions of this article will be made available by the authors, without undue reservation.

## Ethics statement

The studies involving human participants were reviewed and approved by Research Ethics Committee of the New Territories West Cluster, Hospital Authority. The patients/participants provided their written informed consent to participate in this study.

## Author contributions

PL, SL, and CL designed the study protocol. PL and AS undertook the statistical analysis. PL wrote the first draft of the manuscript. DR, AS, SL, and CL commented on the manuscript. All authors contributed to the article and approved the submitted version.

## Conflict of interest

The authors declare that the research was conducted in the absence of any commercial or financial relationships that could be construed as a potential conflict of interest.

## Publisher’s note

All claims expressed in this article are solely those of the authors and do not necessarily represent those of their affiliated organizations, or those of the publisher, the editors and the reviewers. Any product that may be evaluated in this article, or claim that may be made by its manufacturer, is not guaranteed or endorsed by the publisher.

## References

[1] Iyer SN Loohuis H Pawliuk N Joober R Malla AK. Concerns reported by family members of individuals with first-episode psychosis. Early Interv Psychiatry. (2011) 5:163–7. doi: 10.1111/j.1751-7893.2011.00265.x, PMID: 21470375

[2] Ramsay CE Broussard B Goulding SM Cristofaro S Hall D Kaslow NJ. Life and treatment goals of individuals hospitalized for first-episode nonaffective psychosis. Psychiatry Res. (2011) 189:344–8. doi: 10.1016/j.psychres.2011.05.039, PMID: 21708410 PMC3185187

[3] Evensen S Wisløff T Lystad JU Bull H Ueland T Falkum E. Prevalence, employment rate, and cost of schizophrenia in a high-income welfare society: a population-based study using comprehensive health and welfare registers. Schizophr Bull. (2015) 42:476–83. doi: 10.1093/schbul/sbv141 26433216 PMC4753607

[4] Santesteban-Echarri O Paino M Rice S González-Blanch C McGorry P Gleeson J. Predictors of functional recovery in first-episode psychosis: a systematic review and meta-analysis of longitudinal studies. Clin Psychol Rev. (2017) 58:59–75. doi: 10.1016/j.cpr.2017.09.007, PMID: 29042139

[5] Horan WP Green MF DeGroot M Fiske A Hellemann G Kee K. Social cognition in schizophrenia, part 2: 12-month stability and prediction of functional outcome in first-episode patients. Schizophr Bull. (2011) 38:865–72. doi: 10.1093/schbul/sbr001, PMID: 21382881 PMC3406537

[6] Fiske ST Taylor SE. Social Cognition. Second ed. New York: McGraw-Hill (1991).

[7] Green MF Penn DL Bentall R Carpenter WT Gaebel W Gur RC. Social cognition in schizophrenia: An NIMH workshop on definitions, assessment, and research opportunities. Schizophr Bull. (2008) 34:1211–20. doi: 10.1093/schbul/sbm145 18184635 PMC2632490

[8] Pinkham AE Penn DL Green MF Buck B Healey K Harvey PD. The social cognition psychometric evaluation study: results of the expert survey and RAND panel. Schizophr Bull. (2013) 40:813–23. doi: 10.1093/schbul/sbt081, PMID: 23728248 PMC4059426

[9] Bora E Yucel M Panetils C. Theory of mind impairment in schizophrenia: meta-analysis. Schizophr Res. (2009) 109:1–9. doi: 10.1016/j.schres.2008.12.020 19195844

[10] Kohler CG Walker JB Martin EA Healey KM Moberg PJ. Facial emotion perception in schizophrenia: a meta-analytic review. Schizophr Bull. (2010) 36:1009–19. doi: 10.1093/schbul/sbn192, PMID: 19329561 PMC2930336

[11] Combs DR Penn DL Michael CO Basso MR Wiedeman R Siebenmorgan M. Perceptions of hostility by persons with and without persecutory delusions. Cogn Neuropsychiatry. (2009) 14:30–52. doi: 10.1080/13546800902732970, PMID: 19214841

[12] Hoe M Nakagami E Green MF Brekke JS. The causal relationships between neurocognition, social cognition and functional outcome over time in schizophrenia: a latent difference score approach. Psychol Med. (2012) 42:2287–99. doi: 10.1017/S0033291712000578, PMID: 22475159

[13] González-Ortega I González-Pinto A Alberich S Echeburúa E Bernardo M Cabrera B. Influence of social cognition as a mediator between cognitive reserve and psychosocial functioning in patients with first episode psychosis. Psychol Med. (2020) 50:2702–10. doi: 10.1017/S0033291719002794 31637990

[14] Torres US Duran FL Schaufelberger MS Crippa JA Louzã MR Sallet PC. Patterns of regional gray matter loss at different stages of schizophrenia: a multisite, cross-sectional VBM study in first-episode and chronic illness. NeuroImage: Clin. (2016) 12:1–15. doi: 10.1016/j.nicl.2016.06.002, PMID: 27354958 PMC4910144

[15] Eack SM Hogarty GE Cho RY Prasad KM Greenwald DP Hogarty SS. Neuroprotective effects of cognitive enhancement therapy against gray matter loss in early schizophrenia: results from a 2-year randomized controlled trial. Arch Gen Psychiatry. (2010) 67:674–82. doi: 10.1001/archgenpsychiatry.2010.63, PMID: 20439824 PMC3741671

[16] Fisher M Loewy R Hardy K Schlosser D Vinogradov S. Cognitive interventions targeting brain plasticity in the prodromal and early phases of schizophrenia. Annu Rev Clin Psychol. (2013) 9:435–63. doi: 10.1146/annurev-clinpsy-032511-143134, PMID: 23297786 PMC4745413

[17] Yamada Y Inagawa T Sueyoshi K Sugawara N Ueda N Omachi Y. Social cognition deficits as a target of early intervention for psychoses: a systematic review. Front Psychol. (2019) 10:333. doi: 10.3389/fpsyt.2019.00333, PMID: 31156479 PMC6529574

[18] Horan WP Green MF. Treatment of social cognition in schizophrenia: current status and future directions. Schizophr Res. (2019) 203:3–11. doi: 10.1016/j.schres.2017.07.013, PMID: 28712968

[19] Kurtz MM Gagen E Rocha NB Machado S Penn DL. Comprehensive treatments for social cognitive deficits in schizophrenia: a critical review and effect-size analysis of controlled studies. Clin Psychol Rev. (2016) 43:80–9. doi: 10.1016/j.cpr.2015.09.003, PMID: 26437567

[20] Roberts DL Penn DL Combs DR. Unpublished treatment manual. Social cognition and interaction training: University of North Carolina, Chapel Hill, NC (2006).

[21] Roberts DL Penn DL Combs DR. Social Cognition and Interaction Training (SCIT): Group Psychotherapy for Schizophrenia and Other Psychotic Disorders Clinician Guide. (2015) New York: Oxford University Press, PMID: 17293083

[22] Combs DR Adams SD Penn DL Roberts D Tiegreen J Stem P. Social cognition and interaction training (SCIT) for inpatients with schizophrenia spectrum disorders: preliminary findings. Schizophr Res. (2007) 91:112–6. doi: 10.1016/j.schres.2006.12.010, PMID: 17293083

[23] Roberts DL Penn DL. Social cognition and interaction training (SCIT) for outpatients with schizophrenia: a preliminary study. Psychiatry Res. (2009) 166:141–7. doi: 10.1016/j.psychres.2008.02.007, PMID: 19272654

[24] Roberts DL Penn DL Labate D Margolis SA Sterne A. Transportability and feasibility of social cognition and interaction training (SCIT) in community settings. Behav Cogn Psychother. (2010) 38:35–47. doi: 10.1017/S1352465809990464, PMID: 19857363

[25] Roberts DL Combs DR Willoughby M Mintz J Gibson C Rupp B. A randomized, controlled trial of social cognition and interaction training (SCIT) for outpatients with schizophrenia spectrum disorders. Br J Clin Psychol. (2014) 53:281–98. doi: 10.1111/bjc.12044, PMID: 24417608

[26] Penn DL Roberts DL Combs D Sterne A. Best practices: the development of the social cognition and interaction training program for schizophrenia spectrum disorders. Psychiatr Serv. (2007) 58:449–51. doi: 10.1176/ps.2007.58.4.449, PMID: 17412842

[27] Bartholomeusz CF Allott K Killackey E Liu P Wood SJ Thompson A. Social cognition training as an intervention for improving functional outcome in first-episode psychosis: a feasibility study. Early Interv Psychiatry. (2013) 7:421–6. doi: 10.1111/eip.12036, PMID: 23445268

[28] Kanie A Kikuchi A Haga D Tanaka Y Ishida A Yorozuya Y. The feasibility and efficacy of social cognition and interaction training for outpatients with schizophrenia in Japan: a multicenter randomized clinical trial. Front Psychol. (2019) 10:589. doi: 10.3389/fpsyt.2019.00589, PMID: 31507463 PMC6715766

[29] Li Y Sun K Liu D Chen MX Li G Ma J. The effects of combined social cognition and interaction training and paliperidone on early-onset schizophrenia. Front Psychol. (2020) 11:525492. doi: 10.3389/fpsyt.2020.525492, PMID: 33192646 PMC7556232

[30] Wang Y Roberts DL Xu B Cao R Yan M Jiang Q. Social cognition and interaction training for patients with stable schizophrenia in Chinese community settings. Psychiatry Res. (2013) 210:751–5. doi: 10.1016/j.psychres.2013.08.038, PMID: 24018268

[31] Chiao JY Harada T Komeda H Li Z Mano Y Saito D. Dynamic cultural influences on neural representations of the self. J Cogn Neurosci. (2010) 22:1–11. doi: 10.1162/jocn.2009.21192, PMID: 19199421

[32] Abell F Happe F Frith U. Do triangles play tricks? Attribution of mental states to animated shapes in normal and abnormal development. Cogn Dev. (2000) 15:1–16. doi: 10.1016/S0885-2014(00)00014-9

[33] Kessler K Cao L O'Shea KJ Wang H. A cross-culture, cross-gender comparison of perspective taking mechanisms. Proc R Soc B. (2014) 281:20140388. doi: 10.1098/rspb.2014.0388, PMID: 24807256 PMC4024296

[34] Wu S Keysar B. The effect of culture on perspective taking. Psychol Sci. (2007) 18:600–6. doi: 10.1111/j.1467-9280.2007.01946.x 17614868

[35] Dodell-Feder D Ressler KJ Germine LT. Social cognition or social class and culture? On the interpretation of differences in social cognitive performance. Psychol Med. (2020) 50:133–45. doi: 10.1017/S003329171800404X 30616706

[36] Olderbak S Wilhelm O Olaru G Geiger M Brenneman MW Roberts RD. A psychometric analysis of the reading the mind in the eyes test: toward a brief form for research and applied settings. Front Psychol. (2015) 6:1503. doi: 10.3389/fpsyg.2015.01503 26500578 PMC4593947

[37] Hajdúk M Achim AM Brunet-Gouet E Mehta UM Pinkham AE. How to move forward in social cognition research? Put it into an international perspective. Schizophr Res. (2020) 215:463–4. doi: 10.1016/j.schres.2019.10.001, PMID: 31615739 PMC7610500

[38] Bora E Pantelis C. Meta-analysis of cognitive impairment in first-episode bipolar disorder: comparison with first-episode schizophrenia and healthy controls. Schizophr Bull. (2015) 41:1095–104. doi: 10.1093/schbul/sbu198, PMID: 25616505 PMC4535631

[39] Fioravanti M Bianchi V Cinti ME. Cognitive deficits in schizophrenia: an updated metanalysis of the scientific evidence. BMC Psychiatry. (2012) 12:1–20. doi: 10.1186/1471-244X-12-64 22715980 PMC3528440

[40] Deckler E Hodgins GE Pinkham AE Penn DL Harvey PD. Social cognition and neurocognition in schizophrenia and healthy controls: Intercorrelations of performance and effects of manipulations aimed at increasing task difficulty. Front Psychol. (2018) 9:356. doi: 10.3389/fpsyt.2018.00356, PMID: 30131729 PMC6091232

[41] Sergi MJ Green MF Widmark C Reist C Erhart S Braff DL. Social cognition and neurocognition: effects of risperidone, olanzapine, and haloperidol. Am J Psychiatry. (2007) 164:1585–92. doi: 10.1176/appi.ajp.2007.06091515, PMID: 22688151

[42] Lindenmayer JP McGurk SR Khan A Kaushik S Thanju A Hoffman L. Improving social cognition in schizophrenia: a pilot intervention combining computerized social cognition training with cognitive remediation. Schizophr Bull. (2013) 39:507–17. doi: 10.1093/schbul/sbs120, PMID: 23125396 PMC3627756

[43] Lindenmayer JP Khan A McGurk SR Kulsa MKC Ljuri I Ozog V. Does social cognition training augment response to computer-assisted cognitive remediation for schizophrenia? Schizophr Res. (2018) 201:180–6. doi: 10.1016/j.schres.2018.06.012, PMID: 29910120

[44] Pinkham AE Sasson NJ Calkins ME Richard J Hughett P Gur RE. The other-race effect in face processing among African American and Caucasian individuals with schizophrenia. Am J Psychiatry. (2008) 165:639–45. doi: 10.1176/appi.ajp.2007.07101604, PMID: 18347000 PMC7413594

[45] Kay SR Fiszbein A Opler LA. The positive and negative syndrome scale (PANSS) for schizophrenia. Schizophr Bull. (1987) 13:261–76. doi: 10.1093/schbul/13.2.261 3616518

[46] Montgomery SA Åsberg M. A new depression scale designed to be sensitive to change. Br J Psychiatry. (1979) 134:382–9. doi: 10.1192/bjp.134.4.382 444788

[47] Rybarczyk B. Social and occupational functioning assessment scale (SOFAS) In: Kreutzer J Deluca J Caplan B, editors. Encyclopedia of clinical neuropsychology. NY: Springer (2011). 2313. doi: 10.1007/978-0-387-79948-3_428

[48] Gong YX. Chinese version of the Wechsler adult intelligence scale (WAIS-RC). Changsha: Hunan Map Publi. House (1992).

[49] Lo PMT Siu AMH. Assessing social cognition of persons with schizophrenia in a Chinese population: a pilot study. Front Psychol. (2018) 8:302. doi: 10.3389/fpsyt.2017.00302, PMID: 29375405 PMC5767586

[50] Roberts DL Fiszdon J Tek C. Ecological validity of the social cognition screening questionnaire (SCSQ). Schizophr Res. (2011) 37:280–12.

[51] Kanie A Hagiya K Ashida S Pu S Kaneko K Mogami T. New instrument for measuring multiple domains of social cognition: construct validity of the social cognition screening questionnaire. Psychiatry Clin Neurosci. (2014) 68:701–11. doi: 10.1111/pcn.12181, PMID: 24612235

[52] Corcoran R Mercer G Frith CD. Schizophrenia, symptomatology and social inference: investigating “theory of mind” in people with schizophrenia. Schizophr Res. (1995) 17:5–13. doi: 10.1016/0920-9964(95)00024-G, PMID: 8541250

[53] Nuechterlein KH Green MF Kern RS Baade LE Barch DM Cohen JD. The MATRICS consensus cognitive battery, part 1: test selection, reliability, and validity. Am J Psychiatry. (2008) 165:203–13. doi: 10.1176/appi.ajp.2007.07010042, PMID: 18172019

[54] Gordon A Davis PJ Patterson S Pepping CA Scott JG Salter K. A randomized waitlist control community study of social cognition and interaction training for people with schizophrenia. Br J Clin Psychol. (2018) 57:116–30. doi: 10.1111/bjc.12161, PMID: 28990190

[55] Yuen M Gysbers NC Hui EKP Lau PSY Chan RMC Shea PMK. Personal-social development self-efficacy inventory: Users’ manual. A.R: The University of Hong Kong, Hong Kong S (2004).

[56] World Health Organisation. Development of the WHOQOL-BREF from the WHOQOL-100. Geneva: WHO (1997).

[57] Twisk J Bosman L Hoekstra T Rijnhart J Welten M Heymans M. Different ways to estimate treatment effects in randomised controlled trials. Contempor Clin Trials Commun. (2018) 10:80–5. doi: 10.1016/j.conctc.2018.03.008 PMC5898524 29696162

[58] Zhang S Paul J Nantha-Aree M Buckley N Shahzad U Cheng J. Empirical comparison of four baseline covariate adjustment methods in analysis of continuous outcomes in randomized controlled trials. Clin Epidemiol. (2014) 6:227. doi: 10.2147/CLEP.S56554 25053894 PMC4105274

[59] Hawley CJ Gale TM Sivakumaran T Hertfordshire Neuroscience Research group. Defining remission by cut off score on the MADRS: selecting the optimal value. J Affect Disord. (2002) 72:177–84. doi: 10.1016/S0165-0327(01)00451-7 12200208

[60] Healey KM Bartholomeusz CF Penn DL. Deficits in social cognition in first episode psychosis: a review of the literature. Clin Psychol Rev. (2016) 50:108–37. doi: 10.1016/j.cpr.2016.10.001, PMID: 27771557

[61] Kurtz MM Richardson CL. Social cognitive training for schizophrenia: a meta-analytic investigation of controlled research. Schizophr Bull. (2012) 38:1092–104. doi: 10.1093/schbul/sbr036, PMID: 21525166 PMC3446217

[62] Yeo H Yoon S Lee J Kurtz MM Choi K. A meta-analysis of the effects of social-cognitive training in schizophrenia: the role of treatment characteristics and study quality. Br J Clin Psychol. (2022) 61:37–57. doi: 10.1111/bjc.12320, PMID: 34291465

[63] Ludwig KA Pinkham AE Harvey PD Kelsven S Penn DL. Social cognition psychometric evaluation (SCOPE) in people with early psychosis: a preliminary study. Schizophr Res. (2017) 190:136–43. doi: 10.1016/j.schres.2017.03.001, PMID: 28302395 PMC5735418

[64] Davidson CA Lesser R Parente LT Fiszdon JM. Psychometrics of social cognitive measures for psychosis treatment research. Schizophr Res. (2018) 193:51–7. doi: 10.1016/j.schres.2017.06.018, PMID: 28648914 PMC5741542

[65] Durand D Strassnig MT Moore RC Depp CA Ackerman RA Pinkham AE. Self-reported social functioning and social cognition in schizophrenia and bipolar disorder: using ecological momentary assessment to identify the origin of bias. Schizophr Res. (2021) 230:17–23. doi: 10.1016/j.schres.2021.02.011, PMID: 33667854 PMC8222067

[66] Halverson TF Hajdúk M Pinkham AE Harvey PD Jarskog LF Nye L. Psychometric properties of the observable social cognition rating scale (OSCARS): self-report and informant-rated social cognitive abilities in schizophrenia. Psychiatric Res. (2020) 286:112891. doi: 10.1016/j.psychres.2020.112891, PMID: 32145477 PMC7483899

[67] Hermans K Achterhof R Myin-Germeys I Kasanova Z Kirtley O Schneider M. Improving ecological validity in research on social cognition In: Lewandowski KE Moustafa AA, editors. Social cognition in psychosis: London: Academic Press (2019). 249–68.

[68] Zaki J Ochsner K. The need for a cognitive neuroscience of naturalistic social cognition. Ann New York Acad Sci. (2009) 1167:16–30. doi: 10.1111/j.1749-6632.2009.04601.x, PMID: 19580548 PMC2897139

[69] Couture SM Penn DL Roberts DL. The functional significance of social cognition in schizophrenia: a review. Schizophr Bull. (2006) 32:S44–63. doi: 10.1093/schbul/sbl029, PMID: 16916889 PMC2632537

[70] Pinkham AE. Social cognition in schizophrenia. J Clin Psychiatry. (2014) 75:14–9. doi: 10.4088/JCP.13065su1.04 24919166

[71] An SK Kang JI Park JY Kim KR Lee SY Lee E. Attribution bias in ultra-high risk for psychosis and first-episode schizophrenia. Schizophr Res. (2010) 118:54–61. doi: 10.1016/j.schres.2010.01.025, PMID: 20171849

[72] Díaz-Cutraro L García-Mieres H López-Carrilero R Ferrer M Verdaguer-Rodriguez M Barrigón ML. Jumping to conclusions is differently associated with specific subtypes of delusional experiences: An exploratory study in first-episode psychosis. Schizophr Res. (2021) 228:357–9. doi: 10.1016/j.schres.2020.12.037, PMID: 33548835

[73] Dudley R Daley K Nicholson M Shaftoe D Spencer H Cavanagh K. ‘Jumping to conclusions’ in first-episode psychosis: a longitudinal study. Bri J Clin Psychol. (2013) 52:380–93. doi: 10.1111/bjc.12023, PMID: 24117911

[74] Fiszdon JM. Introduction to social cognitive treatment approaches for schizophrenia In: Roberts DL Penn DL, editors. Social cognition in schizophrenia: From evidence to treatment. NY: Oxford University Press (2013). 285–310.

[75] Vaskinn A Horan WP. Social cognition and schizophrenia: unresolved issues and new challenges in a maturing field of research. Schizophr Bull. (2020) 46:464–70. doi: 10.1093/schbul/sbaa034, PMID: 32133507 PMC7147571

[76] Fett AKJ Viechtbauer W Penn DL van Os J Krabbendam L. The relationship between neurocognition and social cognition with functional outcomes in schizophrenia: a meta-analysis. Neurosci Biobehav Rev. (2011) 35:573–88. doi: 10.1016/j.neubiorev.2010.07.001, PMID: 20620163

[77] Bora E Murray RM. Meta-analysis of cognitive deficits in ultra-high risk to psychosis and first-episode psychosis: do the cognitive deficits progress over, or after, the onset of psychosis? Schizophr Bull. (2013) 40:744–55. doi: 10.1093/schbul/sbt085, PMID: 23770934 PMC4059428

[78] Flaaten CB Melle I Bjella T Engen MJ Åsbø G Wold KF. Domain-specific cognitive course in schizophrenia: group-and individual-level changes over 10 years. Schizophr Res: Cogn. (2022) 30:100263 35783460 10.1016/j.scog.2022.100263 PMC9240854

[79] Meier MH Caspi A Reichenberg A Keefe RS Fisher HL Harrington H. Neuropsychological decline in schizophrenia from the premorbid to the postonset period: evidence from a population-representative longitudinal study. Am J Psychiatry. (2014) 171:91–101. doi: 10.1176/appi.ajp.2013.12111438, PMID: 24030246 PMC3947263

