# Supplementary material for: A randomized controlled trial of social cognition and interaction training for persons with first episode psychosis in Hong Kong
Source: Front Psychiatry. 2023 Mar 6;14:1098662. doi: 10.3389/fpsyt.2023.1098662 (PMC10029102; doi:10.3389/fpsyt.2023.1098662)
Supplement: Supplementary file 1 [file Data_Sheet_1.docx]

Table 1

*Demographic and clinical characteristics at T1 (N = 52)*

| Characteristics | SCIT+Rehab  (n = 25) | Rehab  (n = 27) | *p* |
| --- | --- | --- | --- |
| Age, mean (SD) | 25.1 (5.2) | 26.4 (7.5) | .720^a^ |
| Education in years, mean (SD)  Time since psychosis onset, mean (SD) in mths  Male sex  IQ, mean (SD)  Primary Diagnosis  Schizophrenia  Unspecified psychosis  Acute and transient psychosis  Schizoaffective disorder  Secondary Diagnosis  Depression  Anxiety  Suspected pervasive developmental disorder  PANSS_P  PANSS_N  PANSS_G  MADRS  SOFAS  Chlorpromazine equivalents  Taking Artane  Artane dosage, M (SD) in daily mg | 19.9 (22.9)  n = 12, 48.0%  97.4 (19.1)  n = 22, 88.0%  n =2, 8.0%  n = 1, 4.0%  n=0, 0.0%  n=4, 16.0%  n=1, 4.0%  n=0, 0.0%  9.4 (3.9)  12.1(6.2)  21.5 (5.7)  3.8 (7.8)  68.4 (11.8)  448.7 (256.0)  n = 5, 20.0%  5.6 (3.8) | 20.7 (23.3)  n = 11, 40.7%  93.4 (21.7)  n = 23, 85.2%  n = 3, 11.1%  n = 0, 0.0%  n=1, 3.7%  n=4, 14.8%  n=0, 0.0%  n=1, 3.7%  8.1 (2.5)  9.4 (3.4)  19.6 (4.3)  2.6 (3.7)  71.0 (12.9)  439.7 (279.6)  n = 8, 29.6%  4.8 (2.4) | .905  .780^b^  .503  .542^a^  .368 ^a^  .156 ^a^  .082 ^a^  .174  .473  .454  .908  .628 |

Note. ^a^ Mann-Whitney U, ^b^ pearson chi-square OR Fisher’s Exact Test, only the data from participants who were treatment completers (SCIT+Rehab group) and who completed assessment (Rehab group) at follow-up is included.

Table 2

*Comparison of social cognitive and neurocognitive measures between SCIT+Rehab completers and Rehab group at T1 and T2*

| Measures | SCIT+Rehab  (N=28) | | Rehab  (N=31) | | Group x Time interaction  (2 x 2 mixed ANOVA) | | |
| --- | --- | --- | --- | --- | --- | --- | --- |
|  | M (S.D.) | Within-group contrast | M (S.D.) | Within-group contrast | F | p | Partial eta squared |
| Facial Emotion Identification Test (FEIT) | | | | | | | |
| Baseline | 15.57 (3.4) | NS | 15.29 (2.9) | NS | .21 | .65 | .00 |
| Post-treatment | 16.04 (2.8) |  | 15.26 (3.3) |  | (.78) | (.38) | (.01) |
| Social Cognition and Screening Questionnaire (SCSQ) | | | | | | | |
| Theory of Mind (ToM) | | | | | | | |
| Baseline | 6.75 (1.3) | NS | 6.77 (1.6) | NS | .49 | .49 | .01 |
| Post-treatment | 6.46 (1.5) |  | 6.81 (1.2) |  | (1.00) | (.32) | (.02) |
| Attributional Style | | | | | | | |
| Baseline | 1.39 (1.07) | ES = .16* | 1.13 (1.02) | NS | 5.87 | .02* | .09 |
| Post-treatment | 1.02 (.82) |  | 1.34 (1.17) |  | (4.84) | (.03*) | (.08) |
| Jump-to-Conclusion | | | | | | | |
| Baseline | 2.69 (.61) | NS | 2.63 (.87) | NS | 3.28 | .08^+^ | .05 |
| Post-treatment | 2.54 (.40) |  | 2.82 (.68) |  | (5.08) | (.03*) | (.08) |
| MCCB | | | | | | | |
| Speed of Processing | | | | | | | |
| Baseline | 36.87 (11.7) | NS | 36.26 (12.9) | NS | .54 | .47 | .01 |
| Post-treatment | 38.61 (11.4) |  | 39.26 (11.8) |  | (.50) | (.48) | (.01) |
| Attention/Vigilance | | | | | | | |
| Baseline | 44.21 (10.9) | NS | 40.94(12.8) | NS | .25 | .62 | .00 |
| Post-treatment | 43.54 (12.1) |  | 41.29 (13.2) |  | (.07) | (.80) | (.00) |
| Working memory | | | | | | | |
| Baseline | 43.61 (11.7) | ES = .11 ^+^ | 46.71. (10.0) | NS | 2.24 | .14 | .04 |
| Post-treatment | 46.71 (11.6) |  | 45.48 (12.6) |  | (1.30) | (.26) | (.02) |
| Verbal Learning | | | | | | | |
| Baseline | 41.43 (7.8) | NS | 40.48 (9.9) | ES = .12^+^ | .31 | 1.06 | .02 |
| Post-treatment | 43.64 (8.5) |  | 46.19 (14.2) |  | (.89) | (.35) | (.02) |
| Reasoning & Problem Solving | | | | | | | |
| Baseline | 40.57 (13.0) | NS | 37.90 (12.1) | ES = .11^+^ | .02 | .96 | .00 |
| Post-treatment | 43.89 (12.1) |  | 41.35 (10.2) |  | (.22) | (.64) | (.00) |
| Visual Learning | | | | | | | |
| Baseline | 42.82 (13.6) | NS | 41.94 (10.4) | ES = .11^+^ | .02 | .90 | .00 |
| Post-treatment | 45.71 (11.3) |  | 44.55 (10.8) |  | (.09) | (.77) | (.00) |

Note. ES = effect size. NS = not significant; * p < .05; ^+^p < .1 . Baseline score added as covariate shown in blanket.

Table 3

*Comparison of social cognitive and neurocognitive measures between SCIT+Rehab completers and Rehab group at T1, T2 and T3*

| Measures | SCIT+Rehab  (N=25) | | Rehab  (N=27) | | Group x Time interaction  (2 x 3 mixed ANOVA) | | | |
| --- | --- | --- | --- | --- | --- | --- | --- | --- |
|  | M (S.D.) | Within-group contrast | M (S.D.) | Within-group contrast | F | | p | Partial eta squared |
| Facial Emotion Identification Test (FEIT) | | | | | | | | |
| Baseline | 15.48 (3.6) | NS | 15.15 (2.8) | NS | .14 | .84 | | .00 |
| Post-treatment | 15.96 (2.8) |  | 15.15 (3.6) |  | (.36) | (.70) | | (.01) |
| Follow-up | 15.64 (3.3) |  | 15.15 (3.8) |  |  |  | |  |
| Social Cognition and Screening Questionnaire (SCSQ) | | | | | | | | |
| Theory of Mind (ToM) | | | | | | | | |
| Baseline | 6.64 (1.3) | NS | 6.67 (1.7) | NS | .40 | .67 | | .01 |
| Post-treatment | 6.44 (1.5) |  | 6.81 (1.1) |  | (.60) | (.55) | | (.01) |
| Follow-up | 6.64 (1.5) |  | 7.04 (1.7) |  |  |  | |  |
| Attributional Style | | | | | | | | |
| Baseline | 1.48 (1.0) | ES = .15* | 1.14 (1.1) | NS | 3.37 | .04* | | .06 |
| Post-treatment | 1.02 ( .8) | T1>T2* | 1.41 (1.2) |  | (2.35) | (.10) | | (.05) |
| Follow-up | 1.12 (1.2) |  | 1.37 (1.3) |  |  |  | |  |
| Jump-to-Conclusion | | | | | | | | |
| Baseline | 2.68 (.6) | NS | 2.60 (.9) | NS | 2.14 | .12 | | .04 |
| Post-treatment | 2.55 (.4) |  | 2.86 (.7) |  | (2.33) | (.10) | | (.05) |
| Follow-up | 2.49 (.6) |  | 2.77 (1.2) |  |  |  | |  |
| MCCB | | | | | | | | |
| Speed of Processing | | | | | | | | |
| Baseline | 36.08 (12.0) | ES = .16 | 36.22 (13.8) | ES = .19 | .23 | .79 | | .01 |
| Post-treatment | 37.96 (11.6) | T1<T3* | 39.22 (12.5) | T1<T3* | (.26) | (.77) | | (.01) |
| Follow-up | 40.00 (11.3) |  | 41.33 (13.2) |  |  |  | |  |
| Attention/Vigilance | | | | | | | | |
| Baseline | 43.32 (11.1) | NS | 42.15 (12.2) | NS | .07 | .93 | | .00 |
| Post-treatment | 43.60 (12.5) |  | 42.89 (12.9) |  | (.03) | (.97) | | (.00) |
| Follow-up | 44.96 (9.3) |  | 44.63 (13.5) |  |  |  | |  |
| Working memory | | | | | | | | |
| Baseline | 42.44 (11.7) | NS | 45.81 (9.3) | NS | 1.11 | .33 | | .02 |
| Post-treatment | 45.96 (11.9) |  | 44.89 (13.1) |  | (.61) | (.55) | | (.01) |
| Follow-up | 44.84 (12.7) |  | 45.70 (13.6) |  |  |  | |  |
| Verbal Learning | | | | | | | | |
| Baseline | 41.52 (8.1) | ES = .12 | 40.78 (10.5) | ES = .09 | .52 | .60 | | .01 |
| Post-treatment | 42.48 (8.1) | T1<T3^+^ | 45.15 (14.0) | T1<T3^+^ | (.44) | (.65) | | (.01) |
| Follow-up | 45.84 (10.8) |  | 46.78 (13.2) |  |  |  | |  |
| Reasoning & Problem Solving | | | | | | | | |
| Baseline | 40.00 (13.5) | ES = .18 | 37.89 (12.4) | ES = .14 | .28 | .76 | | .01 |
| Post-treatment | 43.88 (12.6) | T1<T3* | 41.00 (10.6) | T1<T3* | (.80) | (.45) | | (.02) |
| Follow-up | 47.44 (12.8) |  | 43.19 (10.2) |  |  |  | |  |
| Visual Learning | | | | | | | | |
| Baseline | 42.16 (14.0) | NS | 41.74 (11.0) | ES = .11 | .10 | .89 | | .00 |
| Post-treatment | 44.92 (11.7) |  | 45.03 (11.4) | T1<T2^+^ | (.11) | (.88) | | (.00) |
| Follow-up | 46.76 (13.8) |  | 45.70 (12.7) |  |  |  | |  |

Note. ES = effect size. NS = not significant; * p < .05 ; ^+^ p < .1. Baseline score added as covariate shown in blanket.

*Table 4.*

*Feedback on SCIT from participants*

|  | Responses | | | | |  |  |
| --- | --- | --- | --- | --- | --- | --- | --- |
| Item | “1”  Totally disagree | “2”  Disagree | “3”  Neutral | “4”  Agree | “5”  Totally agree |  |  |
|  | % of respondents | | | | | M | S.D. |
| 1. To what extent do you agree the content was practical? | 0 | 0 | 10.0 | 66.7 | 23.3 | 4.13 | .57 |
| 2. To what extent do you agree the training has helped you in understanding emotions of other people? | 0 | 0 | 23.3 | 56.7 | 20.0 | 3.97 | .67 |
| 3. To what extent do you agree the training has helped you in understanding thoughts of other people in different social situations? | 0 | 0 | 6.7 | 73.3 | 20.0 | 4.13 | .51 |
| 4. To what extent, do you agree the training has helped you in getting along with people? | 0 | 0 | 23.3 | 43.3 | 33.3 | 4.10 | .76 |
| 5. To what extent, do you agree the participant booklet has helped you in learning the course content? | 0 | 0 | 13.3 | 70.0 | 16.7 | 4.03 | .56 |
| 6. On the whole, are you satisfied with the training? | 0 | 0 | 13.3 | 50.0 | 36.7 | 4.23 | .68 |
